# Diverse environmental bacteria displaying activity against *Phakopsora pachyrhizi*, the cause of soybean rust

**DOI:** 10.3389/fpls.2023.1080116

**Published:** 2023-02-01

**Authors:** Mathias Twizeyimana, Philip E. Hammer, Esther Gachango, Kelly Craig, Billie Espejo, Matthew B. Biggs, James Kremer, David J. Ingham

**Affiliations:** Research and Development, AgBiome, Inc., Research Triangle Park, NC, United States

**Keywords:** microorganism bacteria, biological control, soybean rust, antimicrobial activity, *Phakopsora pachyrhizi*, biocontrol, beneficial microbes, antagonistic effect

## Abstract

The management of soybean rust (SBR) caused by the obligate fungus *Phakopsora pachyrhizi* mostly relies on the use of synthetic fungicides, especially in areas where the disease inflicts serious yield losses. The reliance on synthetic fungicides to manage this disease has resulted in resistance of *P. pachyrhizi* populations to most fungicides. In this study, bacteria isolated from diverse environments were evaluated for their biocontrol potential against *P. pachyrhizi* using soybean detached-leaf method and on-plant in the growth chamber, greenhouse, and field. Among 998 bacterial isolates evaluated using the detached-leaf method; 58% were isolated from plant-related materials, 27% from soil, 10% from insects, and 5% from other environments. Of the isolates screened, 73 were active (they had ⪖ 75% rust reduction) with an active rate of 7.3%. From the active isolates, 65 isolates were re-tested on-plant in the growth chamber for activity confirmation. In the confirmation test, 49 bacteria isolated from plant-related materials maintained their activity with a confirmation rate of 75%. The majority of bacteria with confirmed activity belonged to the taxonomic classes Bacilli and Gammaproteobacteria (70%). Active isolates were prioritized for greenhouse and field testing based on activity in the initial screen and confirmation test. Six bacterial isolates AFS000009 (*Pseudomonas_E chlororaphis*), AFS032321 (*Bacillus subtilis*), AFS042929 (*Bacillus_C megaterium*), AFS065981 (*Bacillus_X simplex_A*), AFS090698 (*Bacillus_A thuringiensis_S*), and AFS097295 (*Bacillus_A toyonensis*) were selected from those bacteria that maintained activity in the confirmation test and were evaluated in the greenhouse, and five among them were evaluated in the field. From the Alabama field evaluation, all bacterial isolates reduced rust infection as well as azoxystrobin (Quadris^®^ at 0.3 L/ha) used as the fungicide control (*P* > 0.05). Moreover, the scanning electron micrographs demonstrated evidence of antagonistic activity of AFS000009 and AFS032321 against *P. pachyrhizi* urediniospores. Bacterial isolates that consistently showed activity comparable to that of azoxystrobin can be improved through fermentation and formulation optimization, developed, and deployed. These bacteria strains would provide a valuable alternative to the synthetic fungicides and could play a useful role in integrated disease management programs for this disease.

## Introduction

Soybean rust (SBR), caused by the obligate fungal pathogen *Phakopsora pachyrhizi* Syd., is considered the most damaging foliar disease of soybean [*Glycine max* (L.) Merr.] in many soybean-growing areas throughout the world. SBR inflicts tremendous yield losses especially when there is lack of serious control measures; losses of up to 80% have been reported in experimental trials in Asia (Hartman et al., 1991), 60% in Brazil and Paraguay (Yorinori et al., 2005), up to 27% in experimental plots and 60% in a commercial field in the U.S. (Mueller et al., 2009; Sikora et al., 2013).

SBR management is achieved primarily through well-timed applications of fungicides (Mueller et al., 2009; Sikora et al., 2014). There are other control methods such as planting resistant cultivars (Twizeyimana et al., 2008) or adopting cultural practices such as staggering the time of planting (Twizeyimana et al., 2011). However, single and dominant resistance genes against *P. pachyrhizi* have shown to be overcome in nature due to the great capacity of the fungus to develop new races (Bromfield, 1984).

Fungicides consisting of triazoles, strobilurins, and their mixtures were found to be the most effective way to manage SBR (Mueller et al., 2009, Godoy, 2012, Sikora et al., 2014). Despite the efficacy of fungicides against this disease, the intensive use of fungicides, especially those with a single mode of action, can result in resistant pathogen populations, hindering management in consecutive crop seasons (Cook, 2001). For instance, some triazole (demethylation-inhibitors, DMI) fungicides were reported ineffective in controlling SBR in fields (Godoy, 2012) and some *P. pachyrhizi* isolates obtained from U.S. samples had high EC_50_ values when treated with azoxystrobin or tebuconazole, indicating a strong likelihood that resistance has started to develop in *P. pachyrhizi* populations in the U.S. (Twizeyimana and Hartman, 2017). The recommendation from the Fungicide Resistance Action Committee (FRAC) is to apply fungicide mixtures exclusively and apply them preventively or as early as possible in the disease cycle, especially when rust pressure is very high and climatic conditions are highly conducive, e.g., prolonged periods of leaf wetness, cool temperatures, etc.

Considering the intensive use of most fungicides, the high risk of fungicide resistance, and the inability to produce resistant soybean cultivars to manage SBR; there is a need to find alternatives to existing control strategies. The use of biocontrol agents may be an important potential solution in the management of SBR and can be deployed in integrated disease management programs for this disease. In general, biological control agents, unlike chemical pesticides, leave behind no long-lasting residues that remain in the environment; they are associated with a low cost of development and a shorter and cheaper registration process (Whipps and Lumsden, 2001). Some of them have unique, complex, and usually, multiple modes of action meaning that pathogens are less likely to evolve into resistance to them (Marrone, 2019).

The most common approach to finding organisms with protective activity consists of isolating microorganisms from samples collected from different environments, selecting antagonistic strains through empirical screening, studying modes of action and formulation of selected microbial isolates (Alabouvette et al., 2006). Antagonistic effects responsible for disease suppression are a result of multiple factors that work alone or in combination. These factors include microbial interactions directed against the pathogen (mainly during its saprophytic phase) or through indirect action that may result in induced resistance of the host plant, and the production of secondary metabolites with antimicrobial properties (Alabouvette et al., 2006; Berg, 2009).

Bacteria or fungi with antimicrobial activity have previously been used as biopesticides for the management of various plant pathogens (Copping and Menn, 2000; Berg, 2009; Srijita, 2015). There have been some reports of bacterial and fungal strains that are antagonistic to *P. pachyrhizi* (Ward et al., 2012; Dorighello et al., 2015) and a few of them were considered potential biocontrol agents for SBR. *Bacillus subtilis* (QST-713) and *B. pumilus* (QST-2808) strains reduced infection caused by *P. pachyrhizi* in detached-leaf, greenhouse, and field experiments (Dorighello et al., 2015). Similarly, strains of *Bacillus* and *Trichoderma* were reported to reduce infection of other rusts, e.g., *B. subtilis* strains AP-3 and AP-150 reduced the number of lesions on the detached leaves of coffee and whole-plant (Bettiol and Várzea, 1992). The fungus *Simplicillium lanosoniveum* was reported to colonize soybean leaves infected with *P. pachyrhizi* in Louisiana and Florida; as uredinia erupted, *S. lanosoniveum* started colonizing urediniospores and eventually killing them. This colonization of urediniospores by *S. lanosoniveum* reduced the development of new uredinia by about fourfold, leading to reduced disease severity under field conditions (Ward et al., 2012).

Bacteria provide a rich resource for the discovery of novel tools to manage plant diseases. Identifying other bacterial microorganisms with high antagonistic activity against *P. pachyrhizi* would lead to the discovery of biocontrol agents that can be formulated and commercially deployed. Additional efficacious products bringing different modes of action can be alternated or mixed with the existing chemicals used in the management of SBR to minimize the development of resistance. Based on the above considerations, the objectives of this study were to (i) determine the biocontrol potential of bacteria isolated from plants, soil, and other environments from AgBiome’s large collection of proprietary environmental isolates against *P. pachyrhizi* using soybean detached-leaf method and on-plant evaluations in the growth chamber, greenhouse, and field; and (ii) study the antagonistic effect of selected bacterial strains against SBR pathogen.

## Materials and methods

### Plant material and inoculum

The SBR-susceptible soybean cv. Williams 82 was used in the initial screen using the detached-leaf assay. The same cultivar was used in the growth chamber at AgBiome, Inc. and in greenhouse evaluations in Florida. Soybean cv. Asgrow 7535 and Pioneer P76T54R2 were used in field evaluations in Fairhope (Alabama) and Quincy (Florida), respectively.

For detached-leaf assays, plants of Williams 82 were planted every three weeks. Briefly, seeds were sown in 18-pot plastic inserts (International Greenhouse Company, Danville, IL, USA) and thinned to one plant per pot after plant emergence. Each insert, with 6 rows of three pots, was filled with soil-less mix (Metro-Mix 360; Sun Gro Horticulture, Bellevue, WA, USA), placed inside a flat (number T1020; International Greenhouse Company, Danville, IL, USA), and fertilized at planting and a month after with slow-release pellets (Osmocote 19-6-12; 1 to 2 pellets/cm^2^). Flats were placed in a growth chamber (Percival Scientific, Inc., Boone, IA, USA) maintained at 75% relative humidity, with a cycle of 13 h of light (150 μmol m^–2^s^–1^ PAR) and 11 h of darkness at 24°C and 23.5°C, respectively, for a constant supply of rust-free soybean leaves (Twizeyimana and Hartman, 2010).

A single-spore isolate of FL07-1 originally collected from infected soybean leaves from Gadsden County, FL in 2007 and multiplied following the process previously described by Twizeyimana and Hartman (2010), was used in detached-leaf and growth chamber evaluations. A mixture of *P. pachyrhizi* urediniospores collected from soybean and kudzu samples in 2015 was used to inoculate greenhouse and field experiments in Florida.

### Bacterial strain isolation

**Figure 1:**
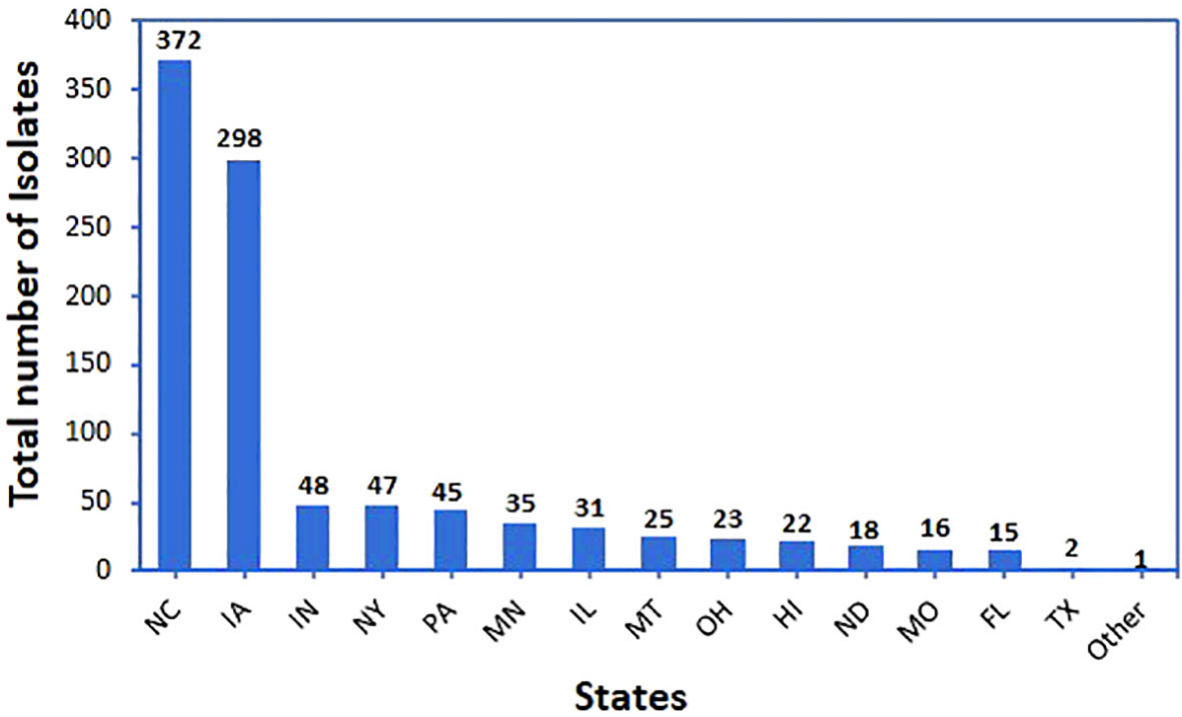
Total number of bacterial isolates and their origin state. All these isolates were evaluated in the initial screen against *Phakopsora pachyrhizi* using a detached-leaf method.

Bacteria were isolated from environmental samples collected from 14 states of the U.S. (**Figure 1** and **Supplementary Figure 1**). They included plant-related materials, soil, insects, and other natural sources. Plant-related materials included leaves (leaf endophyte, phylloplane), roots (root endophyte, rhizoplane), or stems of plants including corn, cucumber, oat, soybean, wheat, and others.

Plant-related materials were processed as follows: (i) phylloplane and rhizoplane samples were collected first; if the latter were covered with excess soil material, they were rinsed with deionized water to remove most soil particles. Each sample was placed in a 50-mL sterile conical tube and approximately 15 mL of 0.1M NaPO_4_ pH7 was added, the tube was vortexed for 1 min, and the resulting phosphate buffer was passed through a 40-μm filter into a new 50-mL sterile conical tube. (ii) Leaf or root tissues in tubes from which phylloplane or rhizoplane samples were collected, or stem tissues were then processed for endophytic samples; briefly, approximately 25 mL of bleach (0.5% NaOCl) was added to the 50-mL conical tube containing leaf, root or stem tissues and mixed for 10 min. The bleach was poured off, the sample was washed with water for 1 min and then washed with 2.5% Na_2_S_2_O_2_ (Sodium Thiosulfate) for 2 min. This was followed by 2 more water washes of 1 min each. The last water wash was poured off and each sample was ground with mortar and pestle. Approximately 15 mL of 0.1M NaPO_4_ pH7 was added during or after grinding and then passed through a 40-μm filter into a new 50-mL conical tube.

For soil, insect, millipede, and worm samples: (i) soil samples were processed by adding soil directly to a 50-mL conical tube (approximately to the 10-mL marker line) and adding 15 mL of 0.1M NaPO_4_ pH7, vortexing for 1 min, and then the resulting phosphate buffer was passed through a 40-μm filter into a new 50-mL conical tube. (ii) For insect, millipede, and worm samples, after being ground in a mortar, they were processed as described earlier for endophytic samples.

The tubes containing the collected filtrate were then spun at 4000 g for 10 min, the supernatant was poured off, leaving ~1 mL of the phosphate buffer behind. The sample was split in half into two 1.8 mL Nunc cryovials. One set was heat-treated at 56°C for 40 min and the other vial was not heated. A total of 250 μL of 40% glycerol was added to each tube and frozen at -80°C.

For bacterial isolation, frozen samples were thawed on ice and 50 μL were plated on an agar medium consisting of 113 mg Na2HPO_4_ 7H2O, 30 mg KH2PO_4_, 10 mg NH4Cl, 100 mg monosodium glutamate, 300 mg molasses, 4.93 mg MgSO4.7H2O, 0.50 mg ZnSO4.7H2O, 0.05 mg FeSO4.7H2O and 15 g Agar per liter of deionized water. Plates were incubated at 25°C for 3-7 days and colony counts were recorded. Individual colonies were streaked in three successive platings to ensure a pure strain (1 single colony morphology) which was subsequently sequenced (full genomic sequencing).

### DNA extraction, genome sequencing and assembly, taxonomic placement, and phylogenetic reconstruction

Bacterial isolates were grown in liquid culture (Biggs et al., 2021), spun down, and DNA was extracted from the cell pellets using MoBio microbial DNA isolation kits (Qiagen, Hilden, Germany). Whole-genome libraries were sequenced on an Illumina HiSeq sequencing platform. Illumina paired-end reads were demultiplexed using bcl2fastq v2.18.0.12 and trimmed using cutadapt version 1.5. Reads were then aligned using CLC Assembly Cell (Qiagen, Hilden, Germany). We calculated genome completeness and redundancy of “essential” single-copy genes—the redundancy of essential, single-copy genes were used to estimate the quantity of contaminating DNA sequence in a given assembly—with CheckM version 1.0.12 (reference data version “2015_01_16“). We used the “bacterial” marker set for all completeness and contamination estimates. Sequences that were contaminated or with poor assembly were not used in this study.

We assessed the taxonomic placement of bacterial strains by placing each strain’s genome assembly in the Genome Taxonomy Database (GTDB) reference taxonomy (Parks et al., 2018) with the GTDB-toolkit version 1.0.2 (Chaumeil et al., 2019). GTDB reconciles historical classifications with whole-genome classifications, and GTDB taxonomic identifiers relate to historical identifiers. For example, the GTDB genus “*Bacillus_A*” is a subset of the historically broad *Bacillus* genus.

**Figure 2:**
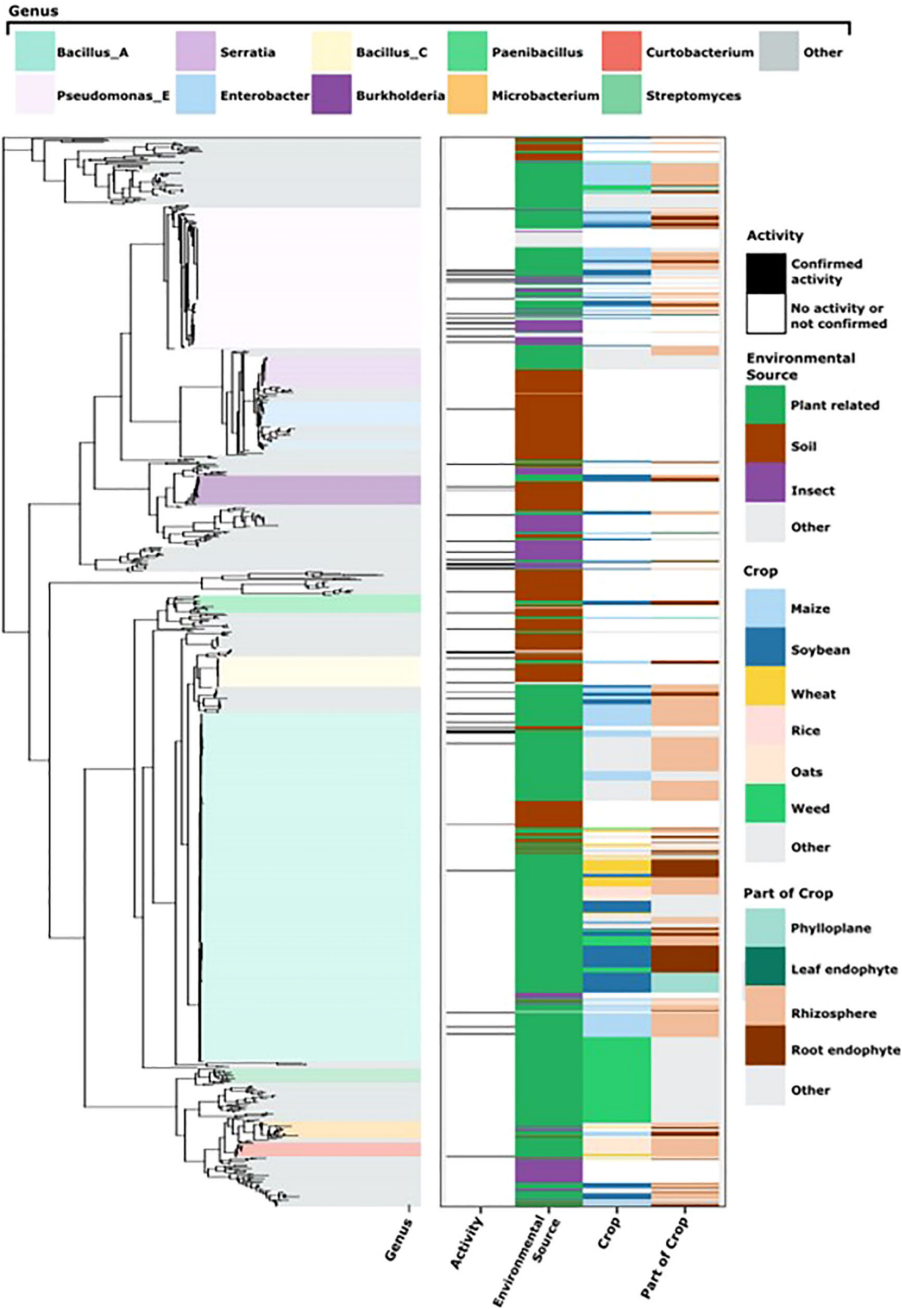
Metadata associated with bacterial isolates evaluated in the initial screen. On the left is the SSU phylogenetic tree with the most abundant genera highlighted as classified by the Genome Taxonomy Database (GTDB) (see key at the top). On the right we indicate activity against soybean rust (at least 75% reproducible rust reduction), environmental source of the isolate, and for plant-associated isolates, the crop, and the part of the crop/plant from which bacteria were isolated (see key to the right).

The SSU rRNA gene sequences were identified and extracted from assembled genomes using the covariance model for the SSU rRNA gene provided by RFam (Griffiths-Jones et al., 2003) (“RF00177”) and Infernal v1.1 (Nawrocki and Eddy, 2013). The longest SSU rRNA gene sequence was selected to represent each strain. Only eighty genome assemblies of 998 possessed more than one SSU rRNA gene. The shortest SSU rRNA gene selected to represent a strain was 1,280 nucleotides. SSU rRNA gene sequences were aligned SSU-Align v0.1.1 (Nawrocki and Eddy, 2013) with a bit-score cutoff of 750 and otherwise default parameters. Alignment positions that were not accounted for in the covariance model were discarded. We further discarded columns where less than 95% of nucleotides had an alignment posterior probability of less than 95%. The final alignment included 1490 positions. We rooted the phylogeny with the SSU rRNA gene sequence from *Sulfolobus islandicus* (Genbank accession: AY247897.1) (Whitaker et al., 2003). The phylogenetic tree (**Figure 2**) was constructed with FastTree version 2.1.10 (general time reversible model for nucleotide evolution) and visualized with ggtree (Yu, 2020).

### Preparation of bacterial strains used in evaluations

For the initial screen and confirmation test, bacterial isolates were retrieved from -80°C and cultured for 2 days in a modified nutrient sporulation medium at 28°C (225 rpm). Modified nutrient sporulation medium consisted of NaCl at 5 g/liter (Thermo Fisher Scientific, Cleveland, OH, USA), tryptone at 10 g/liter (Thermo Fisher Scientific), nutrient broth at 8 g/liter (BD Biosciences), 0.14 mM CaCl_2_ (Sigma-Aldrich, St. Louis, MO. USA), 0.2 mM MgCl_2_6H_2_O (Sigma-Aldrich), and 0.01 mM MnCl_2_4H_2_O (Sigma-Aldrich). Bacterial cells were collected and resuspended in 1 mM MgCl_2_ solution before application to leaf pieces. Colony forming units (CFU/mL) were determined using the standard dilution plate count method, and plates were inspected for signs of contamination. Sterile distilled water was added to achieve the final concentration needed in detached-leaf assays or growth chamber evaluations.

For greenhouse and field evaluation, bacterial strain materials were prepared as follows: culture broth was spun down and the mass of the pellet was determined. For each 100 g of pellet material, 5 g of glycerol was added (5% of the pellet mass). Glycerol was mixed by hand until a uniform consistency was achieved. A total of 20 g (20% by weight of cell paste) of micro-Cel E (Imerys Celite, Roswell, GA, USA) was added to a food processor equipped with a Sabatier blade. Cell paste, glycerol, and micro-Cel E were homogenized briefly into a partially dry crumb-like structure. This end product was spread into aluminum trays and dried at 40°C, normally overnight. Once the product dryness reached a water activity of 0.3 or less, it was milled, screened, and stored at 4°C. Culture titer was performed using the standard dilution plate count method.

### Initial screen and confirmation test

In the initial screen, 998 bacterial strains were evaluated using the detached-leaf technique. Briefly, healthy soybean leaves were excised and cut into uniform leaf disks (35 mm in diameter) using a large cork borer (C.S. Osborne & Co, Harrison, NJ, USA). Leaf disks (each as an experimental unit) were sprayed on the abaxial side with approximately 120 μl of bacterial strain (1 × 10^8^ CFU/mL of sterile distilled water) using a fingertip sprayer (Container & Packaging Supply, Eagle, ID, USA) fitted to a 15 mL conical centrifuge tube (Fisher Scientific, Cat No.14-59-53A). Leaf disks were placed adaxial side down on water-saturated 20 × 20 cm filter paper (Whatman International Ltd., Kent, England) in a transparent plastic box with fitted lid (Blister Box 20 × 20 cm, Placon, Madison, WI, USA); two filter papers were used per box. Each box had 22 leaf disks for bacterial treatments and three other treatments that included a fungicide control (azoxystrobin, Quadris^®^, Syngenta, Greensboro, NC, USA at 0.5 ppm), inoculated-nontreated, and non-inoculated without treatment. Boxes with leaf disks were incubated at room temperature in the dark. Eighteen to 24 hours after treatment, leaf disks were inoculated with a spore suspension of *P. pachyrhizi* urediniospores (approximately 120 μl per leaf disk at 5 × 10^4^ urediniospores/mL of sterile distilled water) using an atomizer attached to air compressor (Twizeyimana and Hartman, 2010). After inoculation, boxes were incubated in the dark for a period of 18 h followed by a cycle of 13 hours of light (40-60 μmol m^-2^s^-1^) at 22.5°C and 11 h of darkness at 21.5°C inside a growth chamber (Percival Scientific, Inc.) maintained at 80% RH. Prior to placing in a growth chamber, boxes were placed inside zip bags (Webster Industries, Peabody, MA, USA). A randomized complete block design was used for this experiment, and each treatment had two replications placed in two different boxes.

Rust severity score for each replication was an average of the number of sporulating uredinia in two arbitrarily selected 1-cm diameter circles of leaf tissue counted 12 to 14 days after inoculation under a dissecting microscope at ×20 magnification (Twizeyimana and Hartman, 2010). The number of sporulating uredinia per each replication was used to calculate percent rust reduction values as follows: 100 − [(number of sporulating uredinia from each treatment/number of sporulating uredinia from the inoculated-nontreated treatment) × 100]. The percent rust reduction value for each treatment was the average of values from both replications.

For the confirmation test, a total number of 65 bacterial strains (**Table 1**) were selected from the initial screen based on their high percent rust reduction values (≥ 75%) and were evaluated on plants in the growth chamber for activity confirmation. In this evaluation, when plants were at V2 growth stage (Plumblee and Harrelson, 2022) the first fully expanded trifoliate leaf was sprayed at the abaxial side with microbial strains or fungicide control (same rate as in the initial screen). Controls described in the initial screen were added. Eighteen to 24 hours after treatment, leaflets were inoculated with *P. pachyrhizi* urediniospore suspension to the abaxial surface as described for the initial screen. After inoculation, plants were maintained in a dew chamber overnight and thereafter placed in a growth chamber maintained at 75% RH with a daily cycle of 13 and 11 h of light (150 μmol m^-2^s^-1^) and darkness at 24 and 22°C, respectively. The design for growth chamber evaluation was a randomized complete block with three replications and the experiment was repeated once. Rust severity data was collected three weeks after inoculation by counting the number of sporulating uredinia in an arbitrarily selected 1-cm diameter circle of leaf tissue from each leaflet of inoculated trifoliate leaves. The percent rust reduction values were calculated as described for the initial screen.

**Table 1:** Percent rust reduction by bacterial isolates selected from the initial screen and re-evaluated on-plant in the growth chamber (confirmation test) for their biocontrol activity against *Phakopsora pachyrhizi*.

Isolate	Taxonomic ID	Environment source	Origin State	Rust reduction (%)^y^	
				Initial Screen	Confirmation Test
AFS092529	*Burkholderia contaminans* ^z^	Soil	NC	95.2	93.5
AFS000009	*Pseudomonas_E chlororaphis*	Plant-related	TX	97.2	93.4
AFS032321	*Bacillus subtilis*	Plant-related	IA	95.5	90.4
AFS011660	*Enterobacter_D kobei*	Soil	IL	87.9	87.7
AFS033812	*Burkholderia cepacia*	Plant-related	NC	94.0	87.5
AFS070623	*Lysobacter enzymogenes*	Soil	NY	76.7	86.4
AFS047845	*Bacillus safensis*	Plant-related	NC	92.2	85.2
AFS036350	*Bacillus_A thuringiensis*	Plant-related	IL	84.3	84.2
AFS076729	*Bacillus_A thuringiensis_J*	Plant-related	NC	87.7	84.0
AFS083363	*Bacillus_C aryabhattai*	Plant-related	NC	97.1	84.0
AFS067990	*Pedococcus* sp.	Plant-related	IL	94.1	83.8
AFS000325	*Cohnella* sp.	Soil	NY	85.2	83.8
AFS065981	*Bacillus_X simplex_A*	Plant-related	IA	92.5	83.4
AFS097295	*Bacillus_A toyonensis*	Plant-related	IL	79.7	83.0
AFS069505	*Paenibacillus* sp.	Insect	IA	83.3	83.0
AFS047008	*Bacillus_X simplex*	Insect	IA	92.5	83.0
AFS082261	*Bacillus_A thuringiensis_J*	Plant-related	NC	76.2	82.7
AFS082943	*Bacillus_A thuringiensis_J*	Plant-related	NC	76.0	82.5
AFS049724	*Burkholderia* sp.	Soil	NC	85.8	81.6
AFS078703	*Paenibacillus amylolyticus_B*	Plant-related	IA	95.3	81.6
AFS086306	*Chromobacterium* sp.	Soil	NC	79.0	81.5
AFS090797	*Bacillus_A thuringiensis_J*	Plant-related	NC	75.5	81.2
AFS002494	*Bacillus_A thuringiensis_S*	Plant-related	IA	89.1	81.2
AFS019430	*Bacillus altitudinis*	Plant-related	NC	82.6	80.8
AFS076171	*Bacillus_A wiedmannii*	Soil	PA	80.9	80.8
AFS043556	*Pseudomonas_E sp900107395*	Soil	–	93.3	80.5
AFS042929	*Bacillus_C megaterium*	Other	IA	83.8	80.5
AFS091268	*Bacillus_A toyonensis*	Plant-related	OH	95.5	80.4
AFS034010	*Streptomyces olivochromogenes*	Insect	NC	81.5	80.3
AFS060579	*Bacillus_X frigoritolerans*	Insect	IA	82.4	79.8
AFS086977	*Pseudomonas_E donghuensis*	Soil	NC	95.3	79.7
AFS046829	*Serratia nematodiphila*	Insect	ND	78.7	79.5
AFS063535	*Bacillus_AW sp001420605*	Plant-related	NC	88.6	79.2
AFS091007	*Burkholderia vietnamiensis*	Plant-related	NC	75.0	79.0
AFS067867	*Pseudomonas aeruginosa*	Other	NC	78.5	78.3
AFS045796	*Bacillus_X frigoritolerans*	Insect	NC	75.7	78.2
AFS090698	*Bacillus_A thuringiensis_S*	Insect	NC	80.3	78.2
AFS047091	*Janibacter melonis*	Plant-related	IA	84.2	78.2
AFS035243	*Mucilaginibacter* sp.	Soil	IA	81.5	77.4
AFS050983	*Mycolicibacterium* sp.	Plant-related	IN	90.5	77.3
AFS047006	*Burkholderia* sp.	Plant-related	NC	87.3	77.0
AFS085990	*Caballeronia jiangsuensis*	Soil	NC	82.5	76.1
AFS059417	*Serratia nematodiphila*	Insect	ND	90.5	76.0
AFS072645	*Paraburkholderia_B*	Soil	NC	80.0	76.0
AFS040381	*Staphylococcus succinus*	Plant-related	IA	83.2	75.8
AFS005289	*Serratia ureilytica*	Insect	ND	75.8	75.7
AFS007963	*Enterobacter asburiae*	Insect	NC	85.7	75.4
AFS040341	*Bacillus* sp.	Other	NC	78.8	75.2
AFS096657	*Agrobacterium tumefaciens*	Plant-related	IA	80.7	75.1
AFS037272	*Burkholderia* sp.	Plant-related	NC	89.5	74.0
AFS079814	*Serratia ureilytica*	Plant-related	IA	77.4	73.8
AFS094304	*Bacillus_A thuringiensis*	Plant-related	MO	76.6	72.8
AFS089684	*Herbaspirillum huttiense*	Plant-related	NC	83.4	72.1
AFS032913	*Serratia nematodiphila*	Insect	ND	76.6	72.0
AFS069057	*Bacillus_A thuringiensis*	Plant-related	MN	84.0	70.5
AFS079521	*Enterobacter sesami*	Plant-related	MO	83.8	70.3
AFS081559	*Thermomonas* sp.	Plant-related	NC	97.2	69.2
AFS030179	*Bacillus_A thuringiensis*	Plant-related	IA	78.1	68.2
AFS097515	*Bacillus_A thuringiensis_J*	Soil	IA	82.2	66.8
AFS082547	*Tsukamurella* sp.	Soil	NY	76.8	64.0
AFS037328	*Bacillus_A thuringiensis_J*	Soil	PA	94.2	59.8
AFS086528	*Bacillus_A thuringiensis_J*	Plant-related	NC	77.1	54.7
AFS008779	*Bacillus_A cereus_T*	Soil	FL	87.5	51.7
AFS008668	*Pseudomonas_E extremorientalis*	Insect	NC	76.2	46.3
AFS030889	*Burkholderia cenocepacia_B*	Corn	IA	81.5	43.3
Fungicide (azoxystrobin at 0.5 ppm)		–	–	95.7	95.0
HSD _0.05_				35.9	26.1

y Percent rust reduction on detached leaves (initial screen) or on leaves of whole plants (confirmation test) treated with different bacterial strains or the fungicide control (azoxystrobin). Percent rust reduction was calculated as follows: [100 - (number of sporulating uredinia/number of sporulating uredinia from the inoculated-nontreated treatment) × 100].

z The bacterial strains were classified using the Genome Taxonomy Database (GTDB).

### Greenhouse and field evaluations

A greenhouse trial was conducted in 2015 at the University of Florida, North Florida Research and Education Center (NFREC) in Quincy, FL. Briefly, seeds of Williams 82 were sown into 22.8-cm-diameter plastic pots containing Metro Mix 300 (Sun GroHorticultural Distributors Inc., Bellevue, WA, USA). Plants were maintained in a rust-free glass greenhouse on metal benches at an average temperature of 26°C and an average RH of 61%. Plants were thinned to one plant per pot after emergence and pots were arranged in a randomized complete block design with three replications.

Treatments included six bacterial strains AFS000009, AFS032321, AFS042929, AFS065981, AFS090698, and AFS097295 (**Table 2**) selected from bacteria for which activity was confirmed on-plant (in the confirmation test), inoculated, non-inoculated, and fungicide controls. Plants at R1 growth stage were sprayed with bacterial strains (7.5 g of formulated product at 50% active ingredient per 1 L of sterile distilled water) and fungicide control (Quadris^®^ at 0.3 L/ha) until runoff using a small hand sprayer. Treated plants were inoculated with a suspension of a mixture of *P. pachyrhizi* urediniospores described earlier (1 × 10^5^ urediniospores/mL of sterile distilled water) until runoff using a hand sprayer a day after treatment application. Rust severity was scored as percent disease severity when plants were at R6 using a nine-point scale which is based on a series of photographs in a booklet entitled “Asian Soybean Rust Disease Severity Evaluation Scale’’ published in 2006 by Bayer CropScience (Research Triangle Park, NC, USA) (Walker et al., 2011).

**Table 2:** Mean numbers of percent rust severity for bacterial isolates evaluated for their activity against *Phakopsora pachyrhizi* in the greenhouse and field trials at North Florida Research and Education Center (NFREC) in Quincy, Florida and in the field trial at the Gulf Coast Research and Extension Center (GCREC) in Fairhope, Alabama.

	% Average rust severity^x^		
Strains	Greenhouse	Field	
	Florida	Alabama^y^	Florida
Azoxystrobin (Quadris^®^ at 0.3 L/ha)	1.3 a	7.6 a	2.2 a
AFS000009 (*Pseudomonas_E chlororaphis*)^z^	6.6 a	10.2 a	5.7 ab
AFS032321 (*Bacillus subtilis*)	5.6 a	13.2 a	4.1 ab
AFS090698 (*Bacillus_A thuringiensis_S*)	8.8 a	10.6 a	8.0 ab
AFS097295 (*Bacillus_A toyonensis*)	8.8 a	15.9 a	9.3 ab
AFS042929 (*Bacillus_C megaterium*)	10 a	15.1 a	4.9 ab
AFS065981 (*Bacillus_X simplex_A*)	11.9 a	–	–
Inoculated (or untreated) control	13.1 a	35.3 b	14.8 b

x Means followed by the same letter within columns are not significantly different at α = 0.05 based on Tukey’s test.

y The field trial in Alabama was not inoculated, rust developed from natural infection.

z The bacterial strains were classified using the Genome Taxonomy Database (GTDB).

The field trials were conducted in 2016 at the Gulf Coast Research and Extension Center (GCREC) in Fairhope, AL (30.522778°N/87.903056°W) and at NFREC-Quincy, FL (30.5427833°N, 84.5956833°W). At GCREC-Fairhope, seeds of Asgrow 7535 were planted at a rate of six seeds per 30 cm within rows. Plots consisted of four 7.6-m long rows with a row spacing of 0.6 m. Plots were separated by 0.9 m and a 5-m long alley separated blocks. At NFREC-Quincy, seeds of Pioneer P76T54R2 were planted at a rate of four seeds per 30 cm within rows. Plots with 8 rows (9.1 m long) with a spacing of 0.25 m were separated by 1.8 m, while blocks were separated by 2.4 m.

In both locations, plants at R1 or R2 growth stage were sprayed (using a backpack sprayer until runoff) with 5 bacterial strains AFS000009, AFS032321, AFS042929, AFS090698, AFS097295 (**Table 2**) and controls as described for the greenhouse study. Treatments were reapplied 14 days after the first application. At NFREC-Quincy, plants were inoculated 1, 4 and 8 day(s) after the first treatment application with a suspension (1 × 10^5^ rust urediniospores/mL of city tap water) of a mixture of *P. pachyrhizi* urediniospores earlier described; in addition to artificial inoculation, natural infection was also present in the field. At GCREC-Fairhope, bacterial strains were evaluated under natural infection. Plots were arranged in a randomized complete block design with four replications. For both field locations, percent disease severity data were recorded using the Bayer CropScience scale (Walker et al., 2011) when soybean plants were at R6 or R7 growth stage. Three individual leaflets at the bottom, middle, and upper layers of 10 randomly selected plants from the two middle rows in each plot were rated individually. Percent disease severity of the entire plant was based on the mean severity of the three canopy levels. The mean of 10 plants was used for each replication.

### Antagonistic effect of *Bacillus subtilis* (AFS032321) and *Pseudomonas_E chlororaphis* (AFS000009) against *Phakopsora pachyrhizi* urediniospores

Using the scanning electron microscopy, the antagonistic activity of bacterial strains AFS00009 and AFS032321 was studied. Their selection was based on their high activity against *P. pachyrhizi* observed in most evaluations. In this experiment, bacterial strain application, inoculation and incubation were conducted as described in the detached-leaf assay. Four treatments were included, (i) leaf disks treated with bacterial strain and inoculated, (ii) treated and non-inoculated, (iii) inoculated without bacterial treatment, and (iv) non-inoculated and no bacterial treatment. Leaf disks (two per each treatment) were collected at different times, 6 hours, 3 days, and 9 days after inoculation. For each leaf disk, a section of approximately 3 mm^2^ was cut using a single-blade straight edge razor and was placed into glass scintillation vials containing approximately 5 mL of fixation buffer with the abaxial surface facing up. Scanning electron micrographs were taken for each leaf section using Field Emission Scanning Electron Microscope – FEI Verios 460L (Analytical Instrumentation Facility (AIF), North Carolina State University, USA) and were colored using the GNU Image Manipulation Program (GIMP, ver. 2.8.14). Micrographs were observed and used in treatment comparison.

### Data analysis

Percent rust reduction values calculated using sporulating uredinia (the formula described earlier) from the initial screen and confirmation test were transformed using arcsine transformation before the analysis of variance (ANOVA) using PROC GLM in SAS (version 9.4; SAS Institute Inc., Cary, NC, USA). Prior to the ANOVA analysis, the homogeneity of variance between confirmation test repeats was tested using Bartlett’s test in SAS. As no heterogeneity was detected, repeats were pooled together and analyzed. Means of percent rust reduction values from the initial screen and confirmation test were compared using Tukey’s test at α = 0.05. Similarly, percent disease severity data from the greenhouse experiment were analyzed using PROC GLIMMIX (biologicals and controls treatments were treated as fixed effects, and replicates as a random effect). Pairwise comparison of treatment effects was conducted using Tukey’s test at α = 0.05.

The effect of biological treatments on percent disease severity was analyzed using PROC GLIMMIX. Treatments (biologicals and controls) were treated as fixed effects, while blocks were treated as a random effect. The statistical analysis was performed separately for each location after preliminary analyses showed differences between both locations. Tukey’s test at α = 0.05 was used for pairwise comparison of treatment effects.

Pearson’s correlation (PROC CORR) was used to establish the relationship between transformed percent rust reduction values from the initial screen and the confirmation test. Similarly, using actual disease severity data, PROC CORR was used to establish the relationship among initial screen, confirmation test, greenhouse, and field evaluations.

## Results

### Microbial strain isolation and characterization

Among 998 bacterial strains evaluated in the initial screen, the highest percentage of strains was isolated from plant-related materials (58%). Others were isolated from soil (27%) and insects (10%), while 5% were from other materials. Maize and soybean were the main plants used in isolations accounting for 48% of bacterial strains isolated from plant-related materials, 31% were isolated from oats, rice, wheat and weeds, whereas 21% were isolated from other plants. Among bacteria isolated from plant-related materials, 48% were from the rhizoplane, 18% from the root endophytic compartment, 8% from leaf endophytic area and 5% from phylloplane, while the remaining 21% were isolated from plant debris or the whole plant (**Supplementary Table 1**).

Bacterial isolates belonged to four phyla, Bacillota with 440 isolates, Pseudomonadota (407), Actinomycetota (129), and Bacteroidota (22). Eight classes represented all bacteria, Bacilli (440), Gammaproteobacteria (263), Actinobacteria (129), Betaproteobacteria (81), Alphaproteobacteria (62), Flavobacteria (15), Sphingobacteria (7), and Cytophagia (1). All bacteria belonged to 126 different genera with *Bacillus* accounting for 38.9%, followed by *Pseudomonas* (13.5%), *Serratia* (3.1%), *Enterobacter* (3.0%), *Paenibacillus* (2.8%), *Burkholderia* (2.7%)*, Microbacterium* (1.6%), and others (31.8%) (**Supplementary Table 1**). There were 324 species representing all bacterial isolates based on Genome Taxonomy Database labels (Parks et al., 2018), (**Figure 2**).

### Initial screen and confirmation test

Based on ANOVA, differences (*P* < 0.0001) in percent rust reduction values were observed among 998 bacterial isolates tested in the initial screen. Among these isolates, 73 (7.3%) exhibited values equal or higher than 75%, 217 (21.7%) showed 50-75% rust reduction and 708 (68%) had values below 50%. In the initial screen or confirmation test, a bacterial isolate was considered to be active (have activity against *P. pachyrhizi*) if it exhibited ≥ 75% rust reduction. We define “active rate” as the percentage of isolates that were active from the initial screen, “confirmation rate” as the percentage of isolates that repeated the activity over the total number of isolates tested in the confirmation test, and “hit rate” as the percentage of isolates with confirmed activity over the total number of isolates screened in the initial screen (these definitions are slightly modified from what was reported by Polgár and Keseru, 2011). Viewing the results from the initial screen perspective, of the 998 isolates screened, 73 were active, resulting in the active rate of 7.3%. In the confirmation test, of the 73 isolates that were active in the initial screen, only 65 were tested. Eight were dropped based on taxonomic similarity or lack of growth to produce enough testing materials. There were differences (*P* < 0.0001) among the 65 bacterial isolates evaluated in the confirmation test. Only 49 isolates had confirmed activity (75% confirmation rate or a hit rate of 5%) (**Table 1**).

**Figure 3:**
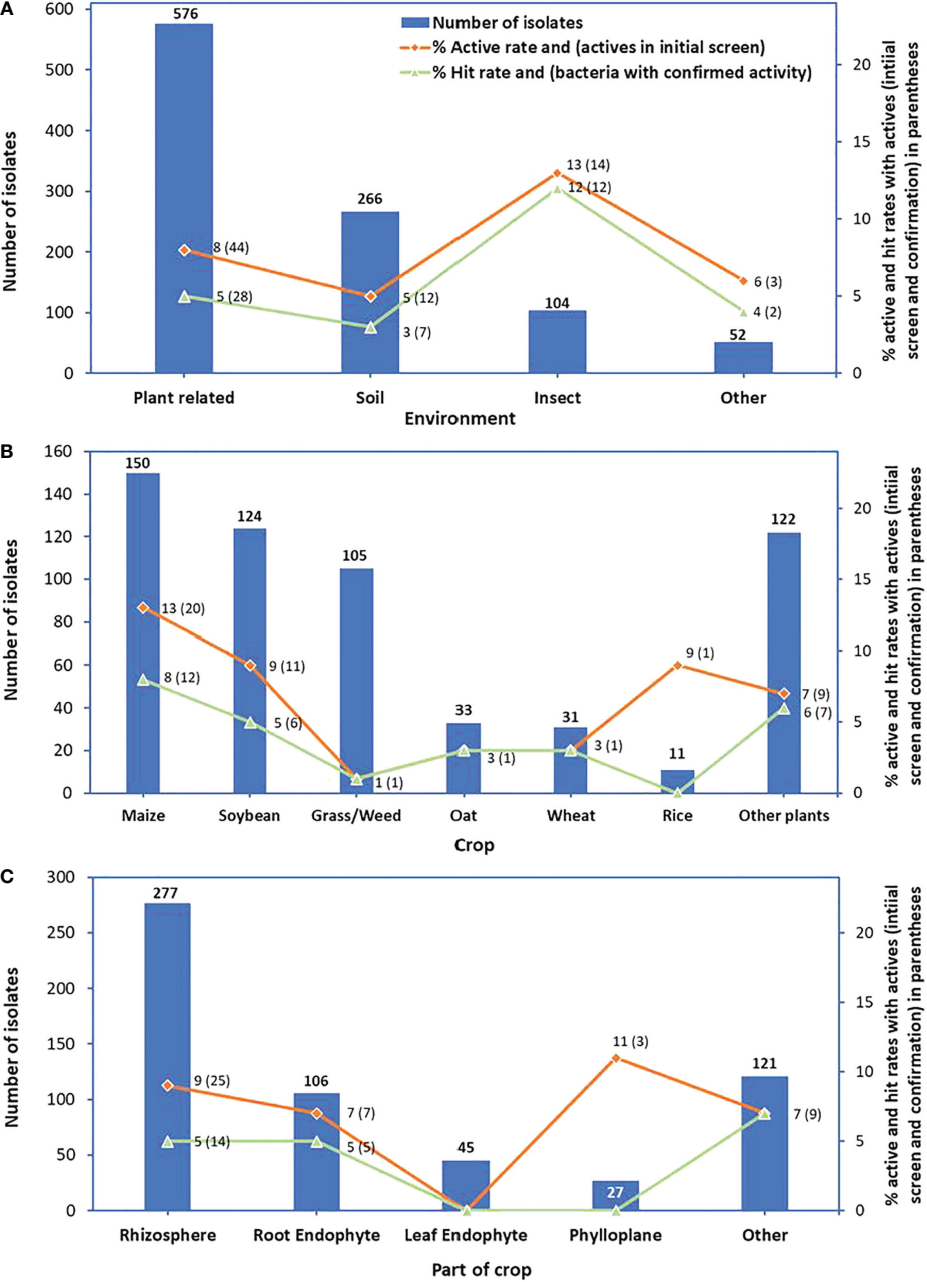
Numbers of bacterial isolates tested in the initial screen (bars), % active rates (red lines) and in parenthesis: numbers of active isolates from the initial screen, and % hit rates (green lines) and in parenthesis: numbers of bacteria with confirmed activity. Graph **(A)** represents the environment, **(B)** crop, and **(C)** part of the crop from which isolates were isolated.

Of the 576 bacterial strains isolated from plant-related materials, 44 were active in reducing rust infection (an active rate of 8%), and 28 strains maintained activity in the confirmation test (a hit rate of 5%). There were 12 bacterial strains among bacteria isolated from the soil that had activity against *P. pachyrhizi* (an active rate of 5%), and 7 bacterial strains maintained activity (a hit rate of 3%). Although the number of bacteria isolated from insects and tested in the initial screen was less than the number of bacteria isolated from plant-related or soil samples, this set had the highest active rate of 13% and the highest hit rate of 12%. Isolates from other materials had the lowest number of active strains (3) with an active rate of 6% and bacteria with confirmed activity (2) with a hit rate of 4% (**Figure 3A**).

For bacteria isolated from plant-related materials, isolates from maize had the highest number of active strains (20) with the highest active rate of 13%. This group of isolates also had the highest number of bacteria with confirmed activity (12) with the hit rate of 8%. Bacteria isolated from soybean had a hit rate of 5%, from grass or weed of 1%, from oat and wheat of 3% each, and from other plants with a hit rate of 6% (**Figure 3B**). Bacteria isolated from the rhizosphere had the highest numbers of active isolates (25) and bacteria with confirmed activity (14); however, their active rate (9%) was lower than that of bacteria isolated from phylloplane (11%) and their hit rate (5%) was lower than that of bacteria isolated from other parts of crop plants (7%) (**Figure 3C**).

**Figure 4:**
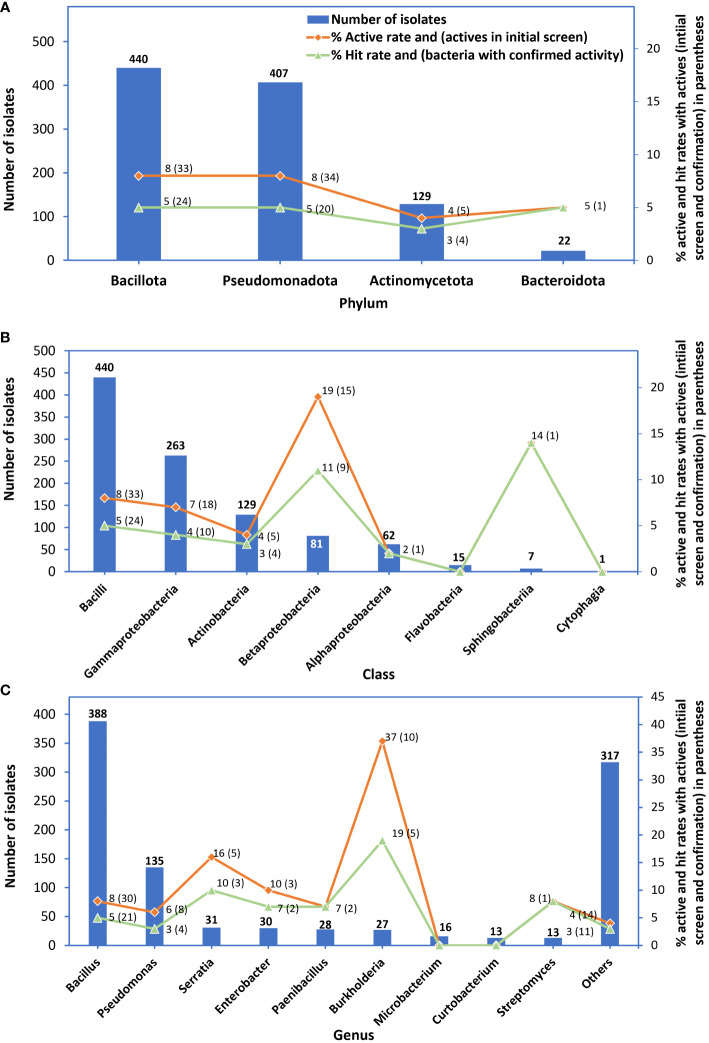
Numbers of bacterial isolates tested in the initial screen (bars), % active rates (red lines) and in parenthesis: numbers of active isolates from the initial screen, and % hit rate (green lines) and in parenthesis: numbers of bacteria with confirmed activity. Graph **(A)** represents phyla, **(B)** class, and **(C)** genus isolates belonged to.

Bacteria belonging to the phylum Bacillota had the highest number of active isolates and bacteria with confirmed activity; however, their active rate (8%) was similar to that of bacteria belonging to the phylum Pseudomonadota and their hit rate of 5% was similar to that of bacteria belonging to the phylum Pseudomonadota or Bacteroidota (**Figure 4A**). Bacterial isolates in the taxonomic class Bacilli had the highest number of active isolates and bacteria with confirmed activity; however, their active and hit rates were lower than those of the class Betaproteobacteria and Sphingobacteria (**Figure 4B**). The genus *Bacillus* had the highest number of active isolates (30) with an active rate of 8% and bacteria with confirmed activity (21) with a hit rate of 5%); however, the hit rate was lower than those of four genera *Burkholderia* (19%), *Serratia* (10%), and *Enterobacter* and *Paenibacillus* (4.1% each) (**Figure 4C**).

### Greenhouse and field evaluations

In the greenhouse evaluation, no significant differences (*P* > 0.05) in percent disease severity were observed among all treatments (**Table 2**), this was likely due to the low disease pressure observed as indicated by the 13.1% disease severity in the inoculated control.

From the GCREC (Fairhope) field evaluation, all bacterial isolates, and the fungicide control (azoxystrobin) had percent disease severity values that were similar (*P* > 0.05) but were significantly lower (*P* < 0.05) than those recorded from the untreated and non-inoculated plot which had the highest mean percent disease severity (35.3%). Similarly, at NFREC-Quincy, all bacterial strains and the fungicide control had similar percent disease severity values (*P* > 0.05) (**Table 2**).

**Figure 5:**
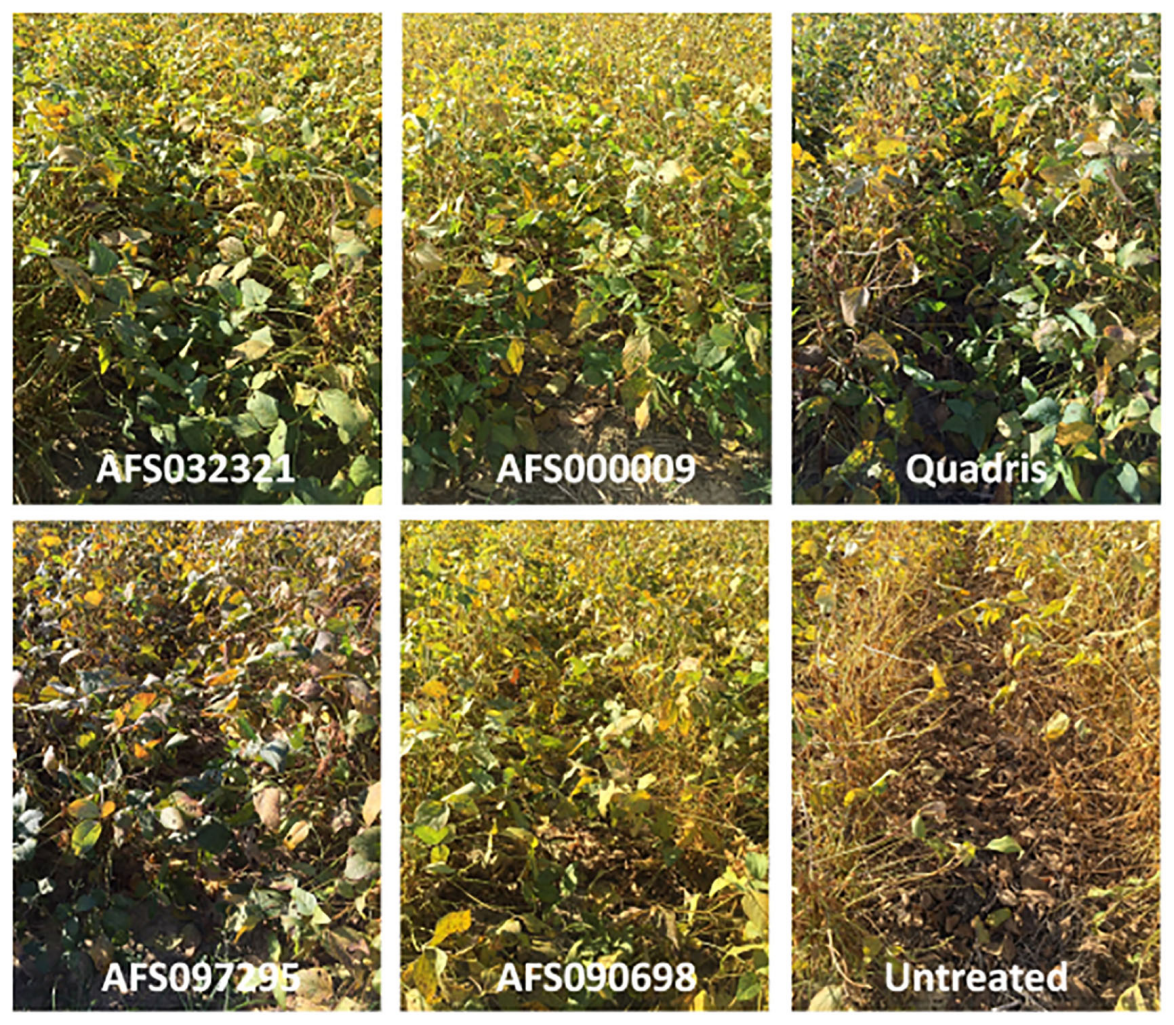
Rust symptoms observed on soybean plants (R6 or R7 growth stages) from the field trial conducted at the Gulf Coast Research and Extension Center (GCREC) in Fairhope, Alabama. Included in this figure are pictures of soybean plots that were treated with four bacterial strains AFS000009 (*Pseudomonas_E chlororaphis*), AFS032321 (*Bacillus subtilis*), AFS090698 (*Bacillus_A thuringiensis_S*), and AFS097295 (*Bacillus_A toyonensis*); the fungicide control azoxystrobin (Quadris^®^ at 0.3 L/ha); and a soybean plot that was not treated (untreated control).

From the field trial at GCREC (Fairhope), rust symptoms observed on soybean plants (R6 or R7 growth stages) treated with bacterial strain AFS000009 or AFS032321 were visually similar to those of soybeans treated with azoxystrobin (Quadris^®^ at 0.3 L/ha). While many green leaves were still visible on soybeans treated with AFS000009, AFS032321, or azoxystrobin at R6 or R7, there was complete defoliation on soybean that were not treated (untreated control) (**Figure 5**).

### Relationship among initial screen, confirmation test, greenhouse, and field evaluations

Pearson’s correlation coefficient indicated a significant positive correlation (*r* = 0.75, *P* < 0.0001) between mean transformed percent rust reduction values recorded in the initial screen and confirmation test (**Table 3**). Similarly, using actual disease severity data there were significant (*P* < 0.05) positive correlations among the initial screen, confirmation test, greenhouse, and field disease severity data. Correlation coefficients ranged from 0.70 to 0.98 (**Table 3**). The highest correlation coefficient was observed between disease severity values in the initial screen and the Field trial at GCREC (Fairhope) (*r* = 0.98, *P* = 0.0001).

**Table 3:** Pearson correlation coefficients from the disease severity data to compare the similarities of initial screen, confirmation test, greenhouse, and field evaluations.

Variables	Initial screen (detached-leaf)	Confirmation Test	Greenhouse (Florida)	Field (Alabama)
**Confirmation Test**	0.75***^yz^	…	…	…
**Greenhouse (Florida)**	0.71*	0.71*	…	…
**Field (Alabama)**	0.98***	0.97***	0.79*	…
**Field (Florida)**	0.90**	0.93**	0.85*	0.87**

y Disease severity data for sixty-seven treatments (65 bacterial isolates, inoculated and fungicide control) were used in the correlation analysis between initial screen and confirmation test data. Eight treatments (6 bacterial isolates, inoculated and fungicide control) were used for the greenhouse evaluation, while 7 (5 bacterial isolates, inoculated and fungicide control) were used for field evaluations in the correlation analysis.

z *, **, and *** = significant at P < 0.05, 0.01, and 0.001, respectively.

### Antagonistic effect of *Bacillus subtilis* (AFS032321) and *Pseudomonas_E chlororaphis* (AFS000009) against *Phakopsora pachyrhizi* urediniospores

**Figure 6:**
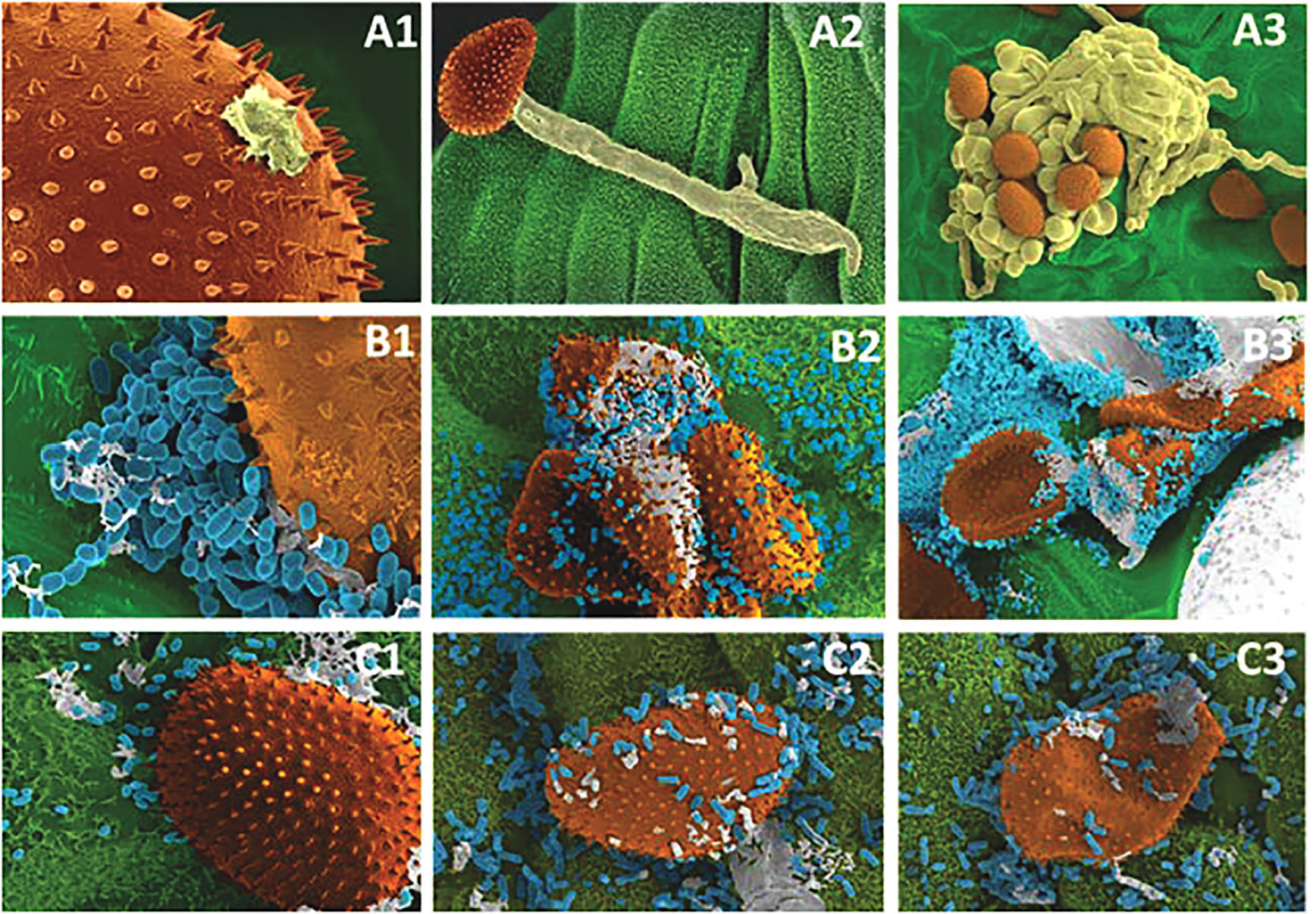
Potential antagonistic effect of *Bacillus subtilis* (AFS032321) and *Pseudomonas_E chlororaphis* (AFS000009) against *Phakopsora pachyrhizi* urediniospores. Healthy urediniospores: **(A1)** urediniospore on the surface of soybean leaf disk showing an area where the germ tube will come out [8,000X], **(A2)** germinated urediniospore with developed germ tube ready to penetrate soybean leaf interior [1,500X], **(A3)** sporulation (9 days after inoculation) [800X]. Bacterial isolates AFS000009 **(B)** and AFS032321 **(C)** colonization: **(B1)** and **(C1)** bacterial cells around urediniospore surface [6,500X], **(B2)** and **(C2)** bacterial cells destroying urediniospores (3 days after inoculation) [2,500], **(B3)** and **(C3)** destroyed urediniospores (deflated, 9 days after inoculation) [2,500].

Scanning electron micrographs demonstrated evidence of potential antagonism activity of AFS000009 and AFS032321 against *P. pachyrhizi* urediniospores. On leaf pieces that were treated with each of these bacteria, cells surrounded urediniospores (**Figures 6B1, C1**), inhibiting germination (**Figures 6B2, C2**), and eventually destroying them a few days after inoculation (**Figures 6B3, C3**). On inoculated leaf pieces which were not treated with bacteria, the urediniospores germinated, infected the leaf, and disease progressed to sporulation 9 to 10 days after inoculation (**Figures 6A1–3**).

## Discussion

This work illustrates a long process involved in the discovery of novel biocontrol agents. The identification of microorganisms with protective activity relies on empirical screening of isolates from samples collected from different environments. In the present study, purified microbial isolates and then fully sequenced to allow characterization and genetic differentiation were evaluated for their biocontrol potential against *P. pachyrhizi*, the cause of SBR. Most bacteria screened (86%) were isolated from plant and soil samples collected from the U.S., with the assumption that plant-related (especially leaf endophyte, phylloplane, rhizosphere, and root endophyte) and soil samples would present a promising source of active microorganisms against *P. pachyrhizi*. Similarly, cook (1993) proposed that the discovery of naturally occurring microbial biocontrol agents for plant diseases and nematodes started with the principle that effective antagonists could be found in local soils or associated with local crop plants (plant-associated microorganisms).

Of the 65 bacterial isolates selected for their activity in the initial screen and re-evaluated on-plant in the confirmation test, 49 maintained the activity. Isolates from plant-related and soil samples were the majority of bacteria with confirmed activity (71%) with a hit rate of 5% and 3% for plant-related isolates and soil, respectively. Although bacteria isolated from insects and other materials had a very small proportion of screened isolates in the initial screen (15% of all isolates) and confirmation test (23%), they accounted for 29% of bacteria with confirmed activity with the highest hit rate of 12% and 4% for the bacteria isolated from insects and other materials, respectively. This suggests that microorganisms with activity against *P. pachyrhizi* can be isolated from any material or environment. Several studies have reported microorganisms (bacteria or fungi) with antimicrobial activity isolated from different environments. For instance, *Bacillus amyloliquefaciens* strain QST 713 (formerly *Bacillus subtilis* strain QST 713), the active strain in Serenade (Bayer Crop Science, USA) or Cease (Bioworks Inc.) was isolated from the soil in a peach tree orchard in Fresno County, California, USA (EFSA, 2021). *Streptomyces* K61 (Mycostop Biofungicide, AgBio, Inc.) was originally isolated from sphagnum peat in Finland (EFSA, 2013), while *B. amyloliquefaciens* subsp. *plantarum* strain D747 (Double Nickel Biofungicide, Certis, USA) was isolated from the atmosphere in Japan (EFSA, 2014).

Viewing the activity in the confirmation test from the crop and part of the crop perspectives, bacteria isolated from maize and soybean had higher hit rates (8 and 5%, respectively). The high likelihood of isolating bacteria with activity against SBR from these crops may be in part explained by the farming practice in most areas of the United States where both crops are traditionally planted in the same fields with soybeans mostly planted after maize. Furthermore, two other crops (oat and wheat) had a hit rate of 3% each, indicating that bacteria that could reduce the infection of this disease might be isolated from other crops. Despite SBR being a foliar disease, most of the bacteria that had confirmed activity were isolated from the rhizosphere and root endophytic compartment (a hit rate of 5% for each). None of the bacteria isolated from leaf endophyte or phylloplane had confirmed activity and this might have resulted from the small number of these bacteria (12.5% of plant-related bacteria) tested in this study. Nevertheless, *Bacillus amyloliquefaciens* (formerly *B. subtilis*) QST 713 strain, the active strain in Serenade or Cease, which is used for the management of several foliar diseases, was isolated from the soil (EFSA, 2021). In contrast, culturing phyllosphere-associated microbes from tomato (Enya et al., 2007) and wheat (Yoshida et al., 2012) resulted in the identification of potential biocontrol microorganisms for foliar pathogens.

As a result of millions of years of cohabitations between bacteria and fungal competitors in many environments, several bacterial lineages have evolved mechanisms to antagonize and exclude fungal pathogens (van Overbeek and Saikkonen, 2016). To find bacteria that can reduce the infection of *P. pachyrhizi* in our study, we screened several bacteria isolated from different environments; however, two phyla with well-known fungicidal activity (Bacillota and Pseudomonadota) had a higher number of screened bacteria and had higher numbers of bacteria that reduced SBR infection in the confirmation test. Similarly, when bacteria belonging to different phyla were screening for their activity against *Colletotrichum sublineola* (sorghum anthracnose) or *Mycosphaerella fijiensis* (black sigatoka), only isolates belonging to two phyla (Bacillota and Pseudomonadota) reproducibly reduced the infection of both diseases (Biggs et al., 2021).

Members of *Burkholderia* and *Serratia* genera had higher % hit rates in this study. Members of both genera have been found to possess antimicrobial compounds active against some plant pathogens (Someya et al., 2001; Hwang et al., 2002; Li et al., 2002; Yu et al., 2022). Despite the beneficial properties, many members of *Burkholderia* (Parke and Gurian-Sherman, 2001) or *Serratia* (Rascoe et al., 2003; Zhang et al., 2005; Petersen and Tisa, 2013) have been reported to be plant pathogens or opportunistic pathogens of humans, making it difficult for members of these genera to be used as active ingredients in commercial biopesticides. Several other genera (*Streptomyces*, *Paenibacillus*, *Enterobacter*, *Bacillus*, and *Pseudomonas*) had % hit rates that ranged from 3 to 8%. Members of these genera have previously been reported to produce antimicrobial compounds. For instance, *Streptomyces* was reported to produce these antimicrobial compounds: streptomycin, pikromycin, kanamycin, nystatin, rapamycin, etc. (Pham et al., 2019), members of the genus *Paenibacillus* are known to be a rich source for antimicrobial compounds useful in the field of agriculture and biotechnology such as antibiotics, enzymes, and other bioactive molecules (Wu et al., 2010; Cochrane and Vederas, 2016), *Enterobacter* spp. were reported to produce siderophores and various antimicrobial compounds, such as bacteriocins, chitinases and antibiotic resistance proteins (Liu et al., 2013). Members of the genus *Bacillus* have been reported to have activity against many plant pathogens (Cook, 1993; Ongena and Jacques, 2008; Choudhary and Johri, 2009; Dorighello et al., 2015). *Bacilli* are known to produce diverse antimicrobial compounds (lipopeptides) such as surfactins, iturins and fengycins which have antagonistic activities for a wide range of potential phytopathogens, including bacteria, fungi, and oomycetes (Ongena and Jacques, 2008; Awan et al., 2023). Three species (*B. amyloliquefaciens*, *B. pumilis*, and *B. subtlis*) are active ingredients of many biofungicide products in the market. Lastly, several compounds (e.g., phenazines, phloroglucinols, pyrrolnitrin, and siderophores) produced by some *Pseudomonas* species have been shown to exhibit both fungistatic and bacteriostatic effects (Dowling and O’Gara, 1994; Win et al., 2022).

Microbial pesticide selection through empirical screening focuses not only on antifungal activity but also on the safety of each strain for non-target organisms. Our high-quality genomic data with full genome coverage enables us to identify bacterial strains which exhibit genetic features indicative of potential plant or animal pathogens (Biggs et al., 2021). The micro-organism and its metabolites must not pose concerns of pathogenicity or toxicity to mammals and other non-target organisms which will likely be exposed to the microbial product (Strauch et al., 2011).

When two bacteria (*B. subtilis* and *P._E chlororaphis*) were further characterized for their antagonism effect against *P. pachyrhizi* urediniospores, micrographs obtained using scanning electron microscopy showed cells of these bacteria surrounding urediniospores, inhibiting germination, and eventually destroying them a few days after inoculation. This corroborated the evidence of antimicrobial properties reported above for some members of *Bacillus* and *Pseudomonas* genera (Cook, 1993; Dowling and O’Gara, 1994; Ongena and Jacques, 2008; Choudhary and Johri, 2009; Dorighello et al., 2015) and the observed activity in our study for both strains against *P. pachyrhizi* in the initial screen, confirmation test, greenhouse, and field evaluations.

The ultimate goal of strain screening is to find a lead strain that may eventually become a commercial product. To accomplish this, thousands of strains must be screened in the initial screen and dozens of strains are retested for activity confirmation. Prioritized strains are subjected to further screenings in the greenhouse and field to shortlist those with the most commercial potential (Bailey and Falk, 2011). In our study, all bacterial isolates prioritized to be tested in the greenhouse and field were grown in generic media with generic fermentation and formulation protocols. To improve the activity of the prioritized bacteria in the greenhouse and field evaluations, the processes such as microbial growth, fermentation, and formulation must be optimized to increase microbial yield and provide a formulated product that is easy to use and deliver high concentration of active materials.

Significant correlations were observed between the initial screen (using a detached-leaf method) and other evaluations including confirmation test (on-plant in the growth chamber), greenhouse, and field disease severity data. This indicates that the initial screening of microbial strains against *P. pachyrhizi* can be carried out effectively and reliably using the detached-leaf assay. This assay allows for simultaneous screening of multiple strains and can significantly speed up the evaluation of new microbial candidates, allowing high-throughput screening of microbial collections. Significant correlations among detached-leaf, greenhouse, and field evaluation data have been previously reported (Foolad et al., 2000; Twizeyimana et al., 2007).

## Conclusion

Currently, fungicides available to manage SBR effectively are limited due to the high propensity of *P. pachyrhizi* to develop resistance to fungicides. Moreover, although several biopesticides have been reported to have activity against SBR in laboratory assays, greenhouse, or field experimental trials, they are not intensively used or included in integrated disease management programs for SBR. The discovery of biologicals with activity comparable to that of synthetic fungicides through empirical screening of bacterial strains and the adoption of integrated disease management measures that include these effective biologicals – used alone and alternating with existing synthetic fungicides or used in mixtures with compatible fungicides – is key to reduction not only of fungicide dependency but also fungicide insensitivity of *P. pachyrhizi* populations, especially in regions where SBR inflicts severe yield losses and its management depends on multiple fungicide applications during one soybean cycle.

## Data availability statement

The datasets presented in this study can be found in online repositories. The names of the repository/repositories and accession number(s) can be found below: https://www.ncbi.nlm.nih.gov/genbank/, Accession numbers OP986005 – OP986995.

## Author contributions

MT and PH designed the overall projects from which data were derived. DI, MB, and JK contributed to the conception and design of experiments. EG, KC, and BE conducted laboratory experiments and collected data. MT and EG analyzed the data. MT drafted the original manuscript. JK and MB added parts to the manuscript. DI, MB, and JK edited the manuscript. All authors contributed to the article and approved the submitted version.
