# Supplementary material for: Diverse environmental bacteria displaying activity against Phakopsora pachyrhizi, the cause of soybean rust
Source: Front Plant Sci. 2023 Feb 1;14:1080116. doi: 10.3389/fpls.2023.1080116 (PMC9932200; doi:10.3389/fpls.2023.1080116)
Supplement: Supplementary file 1 [file Image_1.pdf]

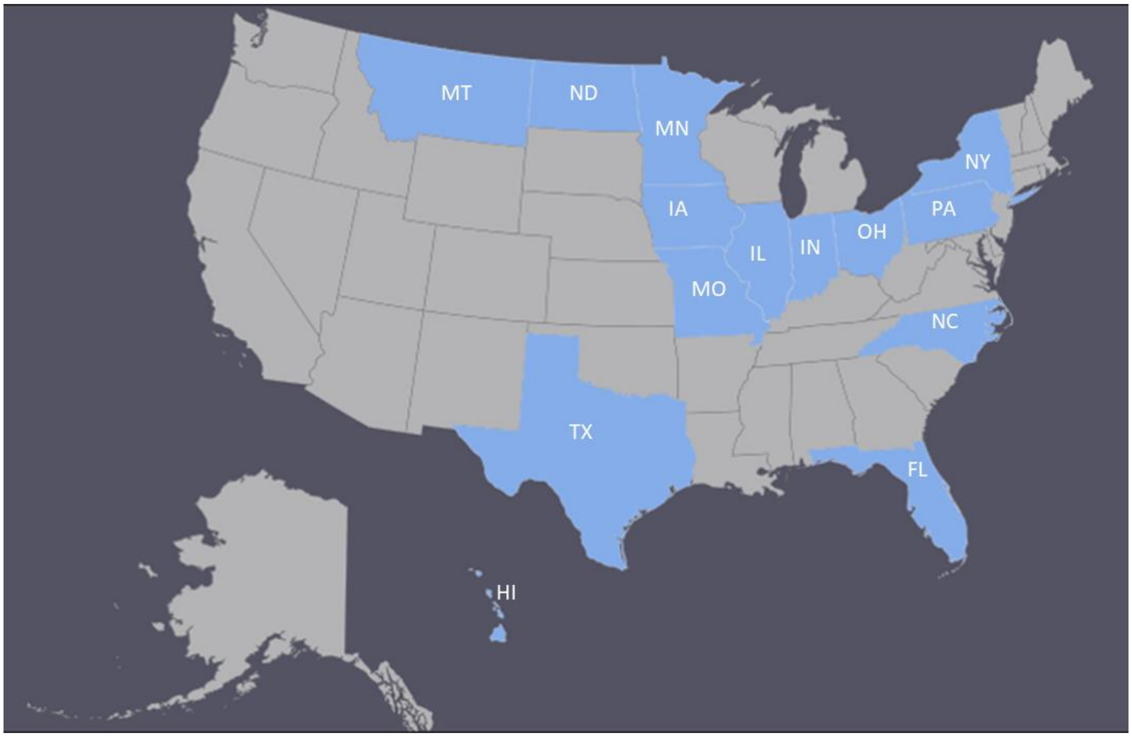

Supplemental Fig. 1. Map of the United States showing states from where samples that produced bacterial strains and evaluated in the initial screen against *Phakopsora pachyrhizi* were collected.
