# Supplementary material for: Diverse environmental bacteria displaying activity against Phakopsora pachyrhizi, the cause of soybean rust
Source: Front Plant Sci. 2023 Feb 1;14:1080116. doi: 10.3389/fpls.2023.1080116 (PMC9932200; doi:10.3389/fpls.2023.1080116)
Supplement: Supplementary file 2 [file Table_1.pdf]

Supplemental Table 1: Numbers of bacterial isolates tested in the initial screen and % of isolates

Environment	Number of isolates	% of isolates
Plant related	576	58
Soil	266	27
Insect	104	10
Other	52	5
Total	998	100

Phylum	Number of isolates	% of isolates
Bacillota	440	44
Pseudomonadota	407	41
Actinomycetota	129	13
Bacteroidota	22	2
Total	998	100

Crop		
Maize	150	26
Soybean	124	22
Grass/Weed	105	18
Oat	33	6
Wheat	31	5
Rice	11	2
Other plants	122	21
Total	576	100

Class		
Bacilli	440	44.1
Gammaproteobacteria	263	26.4
Actinobacteria	129	12.9
Betaproteobacteria	81	8.1
Alphaproteobacteria	62	6.2
Flavobacteria	15	1.5
Sphingobacteriia	7	0.7
Cytophagia	1	0.1
Total	998	100

Part of crop		
Rhizosphere	277	48
Root Endophyte	106	18
Leaf Endophyte	45	8
Phylloplane	27	5
Other	121	21
Total	576	100

Genus		
Bacillus	388	38.9
Pseudomonas	135	13.5
Serratia	31	3.1
Enterobacter	30	3.0
Paenibacillus	28	2.8
Burkholderia	27	2.7
Microbacterium	16	1.6
Curtobacterium	13	1.3
Streptomyces	13	1.3
Others	317	31.8
Total	998	100
